# Active Protein Network Analysis Reveals Coordinated Modules and Critical Proteins Involving Extracellular Electron Transfer Process

**DOI:** 10.3390/genes16060644

**Published:** 2025-05-27

**Authors:** Dewu Ding, Wei Wang, Meineng Wang, Jianming Xie

**Affiliations:** 1 School of Mathematics and Computer Science, Yichun University, Yichun 336000, China; 2 School of Computer Science and Technology, China University of Mining and Technology, Xuzhou 221116, China; 3 School of Biological Science and Medical Engineering, Southeast University, Nanjing 210096, China

**Keywords:** extracellular electron transfer, protein networks, active networks, coordinated modules, critical proteins

## Abstract

Background: Traditional differential expression analysis typically identifies genes with varying expression levels and uses them to construct networks. However, this approach often fails to capture changes in gene interactions that occur at constant gene expression levels. Objectives: To address this limitation, this study investigated the dynamics of protein interactions through active networks under various conditions, focusing on *Shewanella oneidensis* MR-1, a model electroactive microorganism. Methods: We constructed both condition-specific and time-course active protein networks using gene expression and protein interaction data from *S. oneidensis* MR-1. Results: Our analysis revealed several functional modules that were active and well-coordinated under different extracellular electron transfer (EET) conditions. Notably, despite ongoing environmental changes, the dynamics of protein interactions in these networks primarily revolved around two central proteins, SO_0225 and SO_2402. These proteins play crucial roles in coordinating interaction dynamics under oxygen-limited conditions. Additionally, our time-course network analysis elucidated the activation stages of the classical Mtr pathway. Conclusions: This article highlights the dynamic reorganization of protein interaction networks in *S. Oneidensis* MR-1 under varying EET conditions. These findings provide insights into how electroactive bacteria dynamically regulate protein interactions to optimize electron transfer pathways in response to environmental changes.

## 1. Introduction

Under anaerobic conditions, some microorganisms are capable of transferring electrons, produced during their intracellular metabolic processes, to extracellular electron acceptors. This not only generates energy to sustain their growth, but also characterizes the process known as extracellular electron transfer (EET). Accordingly, these microorganisms are referred to as electroactive microorganisms [1]. Due to their unique EET capabilities, these microorganisms have garnered significant attention and have been widely applied in fields such as energy production, wastewater treatment, and chemical synthesis [2,3].

Recent studies have increasingly adopted bioinformatics approaches to explore the molecular mechanisms of EET, aiming to systematically identify key molecules (genes/proteins), elucidate their synergistic effects at the network and pathway levels, and reveal how different EET pathways are selectively activated under various environmental conditions [4,5,6]. Specifically, given the involvement of various molecules in the EET process, researchers have studied the EET process by constructing interaction networks among EET molecules. Zhang and colleagues pioneered this approach with a small-scale network of 18 proteins, examining interactions among key EET proteins like OmcA and MtrC [7]. Sturm and colleagues depicted a dynamic periplasmic electron transfer network and revealed that some periplasmic cytochromes *c* (such as CctA and FccA) could facilitate electron transfer through high-frequency transient protein interactions [8]. They also discovered that the periplasmic electron transfer process involved the allocation of specific EET proteins to particular electron acceptors [8]. To date, significant progress has been made in the study of EET molecular networks [9,10]. Recently, we showed the existence of a highly conserved network motif termed “co-regulated PPI” within integrated transcriptional regulatory and protein interaction networks of *Shewanella oneidensis*. This motif plays a significant role in influencing the “standby” synthesis patterns of proteins, and can be used to identify essential proteins that cells need to synthesize in advance to rapidly respond to environmental changes [11].

Microorganisms typically need to synthesize appropriate proteins to adapt to diverse conditions [12,13]. Accordingly, electroactive microorganisms such as *Geobacter sulfurreducens* and *S. oneidensis* might express different EET molecules under varying environmental conditions, constituting different EET pathways, and forming different network modules. For instance, it has been shown that *G. sulfurreducens* adjusts its metabolism and extracellular respiration in response to different potentials by altering the composition and process of the electron transfer network through differential expression of multiple EET proteins [14].

To better understand these response strategies, we investigated the active response of *S. oneidensis* MR-1 to changes in nutrient conditions, linking transient gene expression to growth phenotypes in the present work. Consistent with previous findings, our research highlights that cellular translation is highly dynamic. Furthermore, we discovered that *Shewanella* employs two hub proteins (SO_0225 and SO_2402) to coordinate interaction dynamics under EET conditions. Specifically, although interactions among proteins in the active network vary with environmental conditions, they predominantly revolve around these two critical proteins, either increasing or disappearing.

## 2. Materials and Methods

### 2.1. Protein Interaction Network

Information on protein interactions was primarily obtained from the STRING database [15,16]. Our analysis began with retrieving interactions for *S. oneidensis* MR-1 from this database. To evaluate the influence of various STRING confidence scores on the results, we applied multiple confidence scores, ranging from 400 (medium confidence) to 700 (high confidence), to filter interactions among proteins based on the combined STRING scores. Additionally, to enhance the reliability of these interactions, we implemented a filter to exclude any protein interactions that lacked direct experimental evidence by using experimental scores greater than 0.

### 2.2. Gene Expression Data

Condition-specific and time-course omics data were used to study the effect of oxygen levels on gene expression in *S. oneidensis* MR-1, particularly in relation to the activation of extracellular electron transfer (EET) processes. Firstly, we used condition-specific mRNA expression data from Ref. [17], which detailed gene expression in *S. oneidensis* MR-1 under both high (20%) and low (8.5%) oxygen conditions. Secondly, we utilized time-course mRNA expression data from Ref. [18], which provided high-throughput RNA sequencing data of samples collected at multiple time points before (as control) and after imposing oxygen limitations (0, 15, 30, 45, and 60 min). Given the critical role of oxygen levels in modulating activation or inactivation of EET pathways, these datasets can serve as valuable resources in understanding how changes in oxygen availability affect the regulatory mechanisms and adaptive responses of electroactive microorganisms.

### 2.3. Active Protein Network

Condition-specific and time-course gene expression profiles are often combined with biological networks to identify sub-networks of activated genes [19]. Sambaturu et al. introduced a computational tool, PathExt, to identify the most dynamic pathways within a protein network [20]. This tool then combined these identified pathways to establish an active protein network (active network), allowing a more focused analysis of pathways particularly active under the condition of interest. Utilizing this approach, we integrated gene expression data for each individual condition with the previously filtered protein network of *S. oneidensis* MR-1 (described in Section 2.1). This integration allowed us to construct condition-specific and time-course active networks, facilitating our analysis of the dynamics of pathway activity under varying conditions.

### 2.4. Enrichment Analysis

We conducted enrichment analysis using the PANTHER protein classification system [21]. Specifically, we employed PANTHER GO-Slim molecular function categories. From these results, we selected only the most specific functional subclasses with a false discovery rate (FDR) of less than 0.05 [22].

### 2.5. Visualization

Visualization of active protein networks was facilitated using Cytoscape 3.9 [23] and NetworkX packages [24].

## 3. Results and Discussion

### 3.1. Highest Activity Networks Were Consistent Under EET Conditions

As the EET process predominantly activates under anaerobic conditions, we analyzed three sets of expression data from low oxygen environments in the literature where the EET process is known to activate [17]. We then combined these expression datasets with the protein networks using multiple confidence scores (combined scores were 400, 500, 600, and 700 and experimental scores greater than 0, as described in Section 2.1). This approach helped us identify three distinct groups of active networks for *S. oneidensis* MR-1 under these low oxygen conditions.

**Figure 1:**
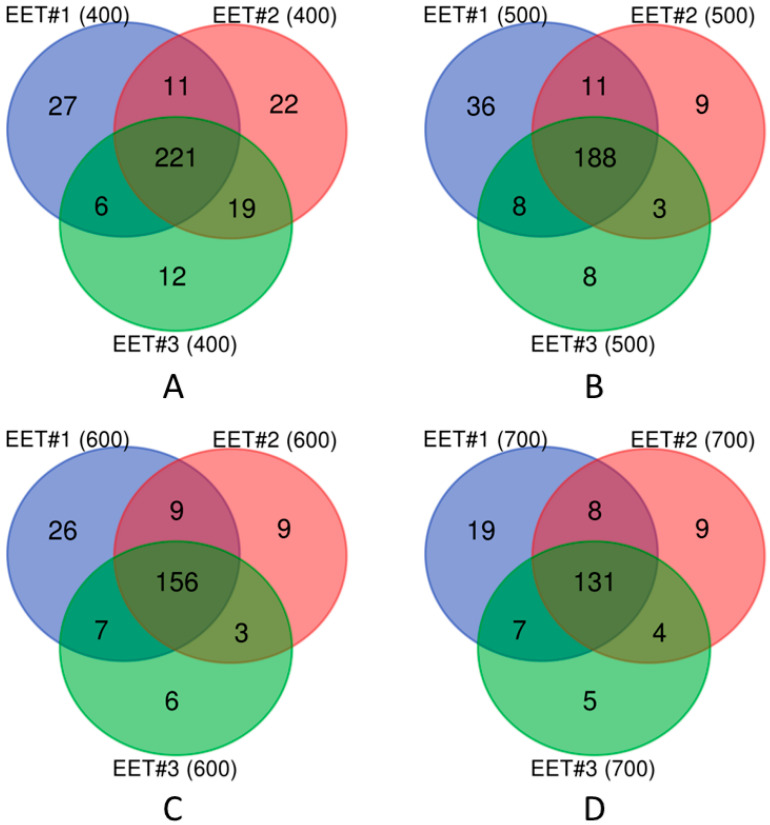
Node comparison of active networks across three EET activation conditions (labeled EET #1, EET #2, and EET #3). Protein–protein interaction (PPI) confidence scores for these networks were (**A**) 400, (**B**) 500, (**C**) 600, and (**D**) 700, respectively.

Further analysis of the node composition of these three groups revealed a significant consistency. As illustrated in Figure 1, regardless of the chosen PPI confidence score, the three active networks shared most of their nodes, with only a few unique nodes across different networks. This indicates a high degree of protein consistency across the highly active networks under the three different EET activation conditions (labeled EET #1, EET #2, and EET #3 in Figure 1).

Notably, the active network under the first EET activation condition (EET #1) consistently exhibited the largest number of unique nodes (Figure 1). In addition, to ensure the reliability of protein–protein interactions, we selected a confidence score of 700. Based on this criterion, the active network EET #1 (700, Figure 1D) was selected for detailed condition-specific active network analysis, as described in the next section.

### 3.2. Condition-Specific Active Networks Revealed Highly Coordinated Modules Under EET Conditions

We first performed a Gene Ontology (GO) enrichment analysis of proteins within the active network. As shown in Table 1, the majority of enriched functions were closely associated with translation processes. These functions included translation elongation factor activity, translation initiation factor activity, structural constituents of ribosomes, rRNA binding, proton-transporting ATP synthase activity, DNA-directed 5′-3′ RNA polymerase activity, protein transmembrane transporter activity, ribosome binding, and mRNA binding, among others. These results indicate that significant changes took place in the cell translation process under activated EET conditions, which may reflect cellular adaptation to meet the energy and protein demands required for efficient electron transfer. These results are consistent with Taylor’s reports [17] and our previous conclusions [25].

**Figure 2:**
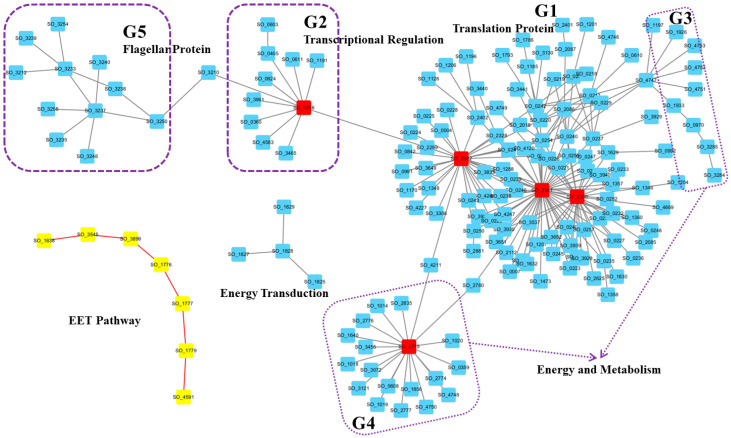
A representative condition-specific active protein interaction network in *S. oneidensis* MR-1 under EET activation. The network was visualized using Cytoscape and highlights several modules (labeled G1 to G5) as well as the Mtr pathway (yellow nodes connected by red edges). The top five hub proteins are marked with red rectangles. Functional categories include translation proteins (G1), transcription regulation (G2), energy and metabolism (G3 and G4), and flagellar proteins (G5). See Supplementary Table S1 for details.

To further examine specific protein compositions and their interactions in this representative active network, we visualized the network using Cytoscape software (Figure 2, Supplementary File S1).

First, we visually distinguished proteins involved in extracellular electron transfer by representing them as yellow nodes connected by red edges (Figure 2). This group includes Mtr pathway-related proteins, which is the most important EET pathway. The inner membrane CymA transfers electrons from the interior to MtrC via MtrA, and then outer membrane cytochrome complex MtrC/OmcA directly mediates electron transfer to extracellular electron acceptors such as Fe(III) [26]. As shown in Figure 2, Mtr pathway-related proteins (CymA, MtrA, MtrC, and OmcA) exhibited high coordination within the active network. The other proteins in this group were outer membrane porin Omp35 (SO_3896), outer membrane porin (SO_3545), and periplasmic chaperone SO_1638 for outer membrane proteins. Previous studies reported that the outer membrane protein Omp35 affects the reduction of Fe(III), nitrate, and fumarate by *S. oneidensis* MR-1 [27]. Furthermore, OmpW (SO_1673), a small outer membrane porin, has also been suggested to facilitate cation transport to maintain electrical neutrality during electron transfer [18]. Based on these reports, we can reasonably assume that the new identified outer membrane porin and chaperone proteins (SO_3545 and SO_1638), which were co-active with and interacted with the Mtr pathway here, may also play an important role in the electron transfer process.

The largest component (Figure 2, G1) was characterized by three hub proteins: SO_2300, SO_2301, and SO_2302. SO_2300 is the translation initiation factor IF-3 (InfC), which can bind to the 30S ribosomal subunit and shifts the equilibrium between 70S ribosomes and their 50S and 30S subunits in favor of free subunits, thus enhancing the availability of 30S subunits on which protein synthesis initiation begins. SO_2301 is the 50S ribosomal protein L35 RpmL, while SO_2302 is the 50S ribosomal protein L20 RplT [28,29]. Moreover, proteins within this cluster are primarily translation function-related proteins, indicating *Shewanella*’s reliance on this large translation protein module to cope with the activated EET conditions. This ensures that *Shewanella* can respond quickly by increasing or altering the production of specific proteins upon EET activation.

In the second group (G2) of proteins within the active network, all components were associated with transcriptional regulation. The hub protein in this module was the DNA-directed RNA polymerase SO_0256, and other proteins were mostly transcriptional activators, transcription elongation factors, transcription termination factors, or RNA polymerases. For instance, the cAMP-responsive regulator Crp (SO_0624) plays a crucial role in anaerobic respiration [30], SspA (SO_0611) acts as a transcriptional activator, and GreA (SO_1191) functions as a transcription elongation factor [31,32]. The proteins in the G3 and G4 groups were mostly associated with energy and metabolism, respectively. For examples, the hub protein SO_2775 was identified as the Acyl carrier protein AcpP, which participates in the fatty acid biosynthesis pathway. These results indicate that in addition to adjustment of the translation process, *Shewanella* also adapts to new environments through alterations in transcription and metabolism.

Finally, the 11 proteins in group G5 were all flagellar-related proteins, and the bridging protein SO_3210 is the RNA polymerase sigma factor for flagellar operons. These results agreed with several recent experimental studies conducted by different research groups that showed flagellar proteins’ involvement in electron transfer activity in *G. sulfurreducens* and *S. oneidensis* (e.g., [33,34,35]). In other words, this highly active network can also imply that flagella proteins play an important role under the activated EET conditions.

### 3.3. Time-Course Active Networks Analysis Revealed Critical Proteins Driving Cellular Translation Dynamics

**Figure 3:**
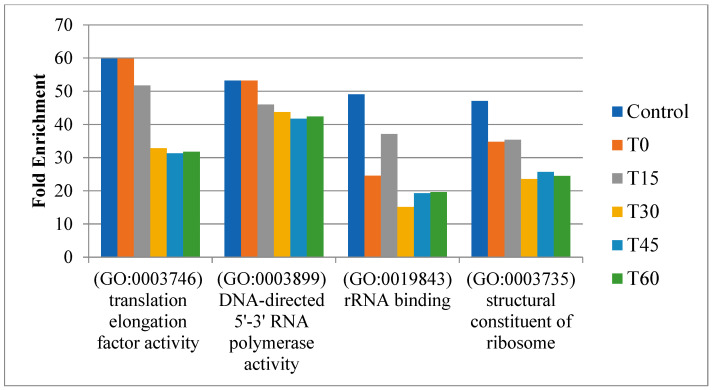
Consistent enriched GO terms for proteins across different stages of active networks.

We integrated time-course expression data from Barchinger et al. [18] with protein networks to construct time-course active networks (a control and five time points: T0, T15, T30, T45, and T60). Next, we examined how these active networks changed throughout different time points. GO enrichment analysis for proteins within these networks showed consistent enrichment across all stages for several GO terms, highlighting a sustained increase in the protein translation activities of *S. oneidensis* MR-1 under oxygen-limited conditions. These terms included translation elongation factor activity (GO:0003746), DNA-directed 5′-3′ RNA polymerase activity (GO:0003899), rRNA binding (GO:0019843), and structural constituents of ribosomes (GO:0003735) (Figure 3). These results clearly indicate that *S. oneidensis* MR-1 needed to coordinate protein translation changes to deal with changed oxygen levels, which is consistent with previous condition-specific active network analysis.

**Figure 4:**
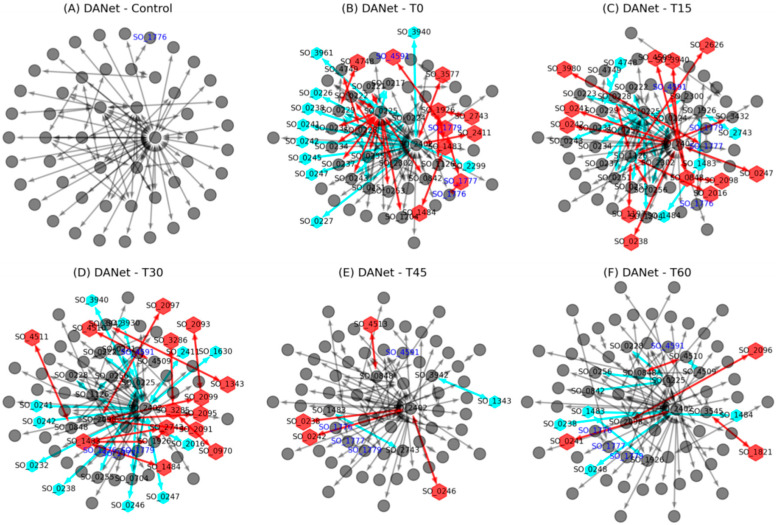
Dynamic changes in time-course active networks of *S*. *oneidensis* MR-1 during EET activation. Comparisons across six stages (control, T0, T15, T30, T45, and T60) revealed temporal shifts in protein composition (red/green nodes indicate new or lost proteins) and interaction patterns (red/green links indicate new or lost interactions). Early stages (T0–T30) showed significant remodeling, while later stages (T45–T60) exhibited stabilization.

Further analysis revealed that while similar biological processes were regulated across different stages, specific proteins and their interactions within these processes varied significantly (Figure 4). Compared to the active network in the previous stage, the majority of proteins were retained across successive stages (gray nodes). Notable changes in protein composition occurred during early stages (T0, T15, and T30), with some proteins newly added (red nodes) or lost (green nodes), and these changes became relatively minor during the T45 and T60 stages. This indicates that, as the EET process was activated, bacteria initially adjusted their protein composition to adapt to this process and gradually stabilize over time. Furthermore, along with changes in proteins in the active networks, significant alterations in protein interactions also occurred (red and blue links), suggesting that microorganisms adapted to changing conditions not only by modifying protein expression, but also by reorganizing interaction patterns.

Although interactions among proteins in the active network were highly dynamic and continually changed in response to environmental conditions, most of these interactions tended to cluster around two critical proteins, SO_0225 and SO_2402 (Figure 4). Specifically, during the T0 stage, SO_0225 added more than ten interactions, while SO_2402 experienced a reduction in similar interactions. This pattern reversed at the T15 stage, with SO_2402 gaining over ten interactions and SO_0225 losing a similar number. By the T30 stage, many of these interactions involving SO_2402 had disappeared again. The active network then tended to stabilize during the T45 and T60 stages, with few changes overall. These findings suggest that a “rapid response–gradual stabilization” protein interactions reorganization strategy was employed by *S. oneidensis* MR-1 to adapt to environmental changes. This pattern is similar to the gene-level competition for protein synthesis capacity within bacterial cells [36], indicating that such competition also occurs at the level of protein interactions. Such a dynamic adjustment strategy may reflect cellular strategies for allocating proteomic resources under limited resources conditions.

As there are currently no literature reports on the biological functions of proteins SO_0225 and SO_2402, the structural information for these two proteins is not available in the PDB. To better understand the functions of SO_0225 and SO_2402, we predicted the structures of these two proteins using the SWISS-MODEL Repository (SMTL Version 2025-05-07) [37]. Results show that the best homolog of SO_0225 is 8f1j.1 (sequence identity 80.77%), which is an early melting intermediate of RNA polymerase capable of binding to mismatched DNA fragments, while the best homolog of SO_2402 is 9gut.1 (sequence identity 84.30%), which is a 30S mRNA delivery complex. This suggests that these two proteins play important roles in transcription initiation and mRNA delivery, respectively.

In total, supplementary to previous findings, our results highlight that cellular responses are highly dynamic. *Shewanella* adapt to changing conditions by adjusting both proteins and their interactions, and two critical proteins (SO_0225 and SO_2402) play crucial roles in coordinating interaction dynamics under oxygen-limited conditions in order to better adapt to new environments.

Furthermore, it is noteworthy that since its activation at the T0 stage, the Mtr pathway was continuously active, as indicated by the blue text nodes in Figure 4. This demonstrates the essential role of this EET pathway in enabling bacteria to transfer electrons from inside the cell to the external environment under oxygen-limited conditions. Our focus here was on elucidating the dynamics of this classic EET pathway.

**Figure 5:**
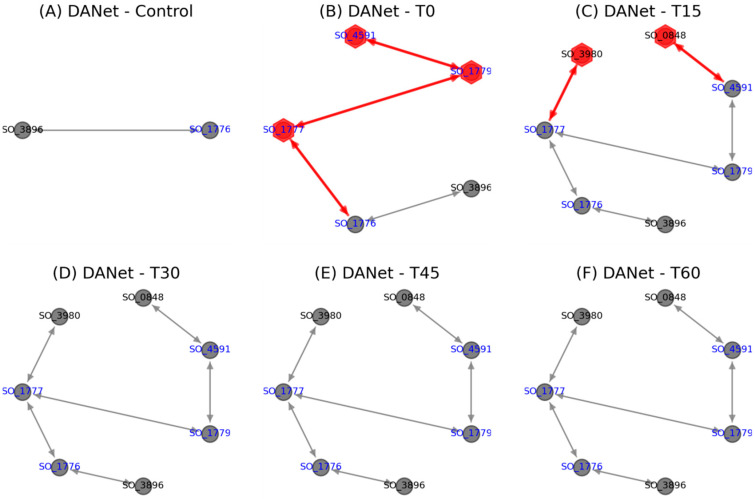
The activation process of the Mtr pathway in *Shewanella* (red nodes/links indicate new added proteins/protein interactions). This figure also shows the later participation of nitrate reductase NapA (SO_0848) and nitrite reductase NrfA (SO_3980).

Further investigations into the activation process of this EET pathway (Figure 5) revealed that the extracellular iron oxide respiratory system outer membrane component MtrB was present even before the pathway was activated. Upon the onset of changed conditions at the T0 stage, key components, including the inner membrane protein CymA (SO_4591), MtrA (SO_1777), and the outer membrane protein OmcA (SO_1779), were immediately activated and remained consistently active, affirming the Mtr pathway as the main route for electron transfer under the studied conditions. CymA acts as an inner membrane electron transfer hub, transferring electrons from the quinone pool to the periplasmic MtrA, and ultimately exporting them to the extracellular space via MtrC [26]. Subsequently, at the T15 stage, the addition of nitrate reductase NapA (SO_0848) and nitrite reductase NrfA (SO_3980) indicated their participation in this process. This involvement, unchanged in subsequent stages, suggests potential cooperation between the Mtr pathway and these cytochromes under specific conditions. Notably, that the Escherichia coli CymA orthologue NapC also exhibits ferric reductase activity [38] supports this hypothesis, pointing to a synergistic interaction that may be characteristic of *Shewanella* under certain environmental pressures. These insights call for further experimental verification to understand the full extent of these interactions and their functional implications.

## 4. Conclusions

In this study, we constructed condition-specific and time-course active protein networks to explore synergistic effects among EET-related molecules and the selective activation of EET pathways.

Our condition-specific network analyses identified several highly coordinated functional modules (G1–G5) that were specifically activated under EET-inducing conditions. These modules include translation-related proteins (G1), transcriptional regulation (G2), energy and metabolism (G3 and G4), and flagellar proteins (G5). Each module plays a distinct role in facilitating the cell’s adaptive response to environmental changes under active EET conditions. For example, translation-related modules ensure rapid synthesis of necessary proteins upon EET activation, while flagellar proteins were shown to be involved in electron transfer activity. Therefore, these modules not only highlight the complexity of cellular adaptation during EET, but also reveal how different biological processes are coordinately regulated to support efficient electron transfer. Although direct experimental validation was beyond the scope of this work, key hub proteins within each module (e.g., SO_2300-SO_2302 in G1) represent promising candidates for future functional studies.

Furthermore, time-course network analysis explored dynamic changes in active protein networks across six time points. During the early stages (T0–T30), significant remodeling was observed, while the later stages (T45–T60) showed increasing stability, revealing a “rapid response–gradual stabilization” pattern in the reorganization of protein interactions in response to environmental fluctuations, with SO_0225 and SO_2402 emerging as two critical proteins. We also presented the activation process of the classical Mtr pathway in *Shewanella*.

Overall, our network-based analysis provides novel insights into identifying key EET molecules in electroactive microorganisms. These findings not only deepen our understanding of microbial extracellular electron transfer, but also offer opportunities to select highly efficient strains for electricity production, as well as to enhance electron transfer efficiency through genetic engineering.

## Figures and Tables

**Table 1:** GO molecular function enrichment for proteins in the active network EET #1 (700). Note: false discovery rate (FDR) < 0.05.

Molecular Function	Number	Expected	Enrichment Fold	FDR	Genes
translation elongation factor activity (GO:0003746)	4	0.16	24.65	5.01 × 10^−5^	*SO_0217*, *SO_0229*, *SO_1630*, *SO_2328*
translation initiation factor activity (GO:0003743)	2	0.08	24.65	1.93 × 10^−2^	*SO_1204*, *SO_2300*
structural constituent of ribosome (GO:0003735)	38	1.58	24.02	8.67 × 10^−52^	*SO_0220*, *SO_0222*, *SO_0223*, *SO_0226*, *SO_0227*, *SO_0230*, *SO_0231*, *SO_0232*, *SO_0233*, *SO_0234*, *SO_0235*, *SO_0236*, *SO_0237*, *SO_0238*, *SO_0240*, *SO_0241*, *SO_0243*, *SO_0244*, *SO_0245*, *SO_0246*, *SO_0248*, *SO_0250*, *SO_0255*, *SO_0257*, *SO_1357*, *SO_1360*, *SO_1629*, *SO_2301*, *SO_2302*, *SO_2402*, *SO_3651*, *SO_3652*, *SO_3928*, *SO_3930*, *SO_3939*, *SO_3940*, *SO_4246*, *SO_4247*
rRNA binding (GO:0019843)	10	0.49	20.55	2.90 × 10^−11^	*SO_0220*, *SO_0227*, *SO_0238*, *SO_0241*, *SO_0247*, *SO_0255*, *SO_2112*, *SO_3537*, *SO_3928*, *SO_3930*
proton-transporting ATP synthase activity, rotational mechanism (GO:0046933)	4	0.2	19.72	2.04 × 10^−4^	*SO_4746*, *SO_4748*, *SO_4749*, *SO_4750*
DNA-directed 5′-3′ RNA polymerase activity (GO:0003899)	2	0.12	16.44	4.58 × 10^−2^	*SO_0224*, *SO_0225*
protein transmembrane transporter activity (GO:0008320)	2	0.12	16.44	4.31 × 10^−2^	*SO_0218*, *SO_0251*
ribosome binding (GO:0043022)	6	0.53	11.38	9.95 × 10^−5^	*SO_1170*, *SO_1346*, *SO_1632*, *SO_1793*, *SO_2300*, *SO_2625*
mRNA binding (GO:0003729)	4	0.41	9.86	6.04 × 10^−3^	*SO_0223*, *SO_0227*, *SO_2402*, *SO_3940*
NADH dehydrogenase activity (GO:0003954)	3	0.32	9.25	3.34 × 10^−2^	*SO_1014*, *SO_1018*, *SO_1020*

## Data Availability

All data generated and analyzed during this study are included in this article.

## Supplementary Materials

The following supporting information can be downloaded at: https://www.mdpi.com/article/10.3390/genes16060644/s1. Supplementary File S1. Cytoscape file of the active protein interaction network shown in Figure 2. Supplementary File S2. Supplementary Table S1: Functional annotation and module classification (G1–G5) of proteins in the network shown in Figure 2.
